# Asymptomatic SARS-COV2 Infection or COVID-19 vaccination effect for severe multisystem inflammatory syndrome in a 6-year-old girl: case report and review of the literature

**DOI:** 10.1186/s13052-024-01758-y

**Published:** 2024-09-27

**Authors:** Enrica Manca, Michele di Toma, Marianna Esotico, Lucia Soldano, Anna Nunzia Polito, Giuseppina Mongelli, Leonardo Guglielmi, Francesca Scaltrito, Angelo Campanozzi

**Affiliations:** 1 Pediatrics Department, University Hospital of Foggia, Foggia, Italy; 2 IDESP, UA11-INSERM, University of Montpellier, Montpellier, France; 3 University of Foggia, Foggia, Italy; 4 Department of Woman and Child, Neuropsychiatry for Child and Adolescent Unit, University Hospital of Foggia, Foggia, Italy

**Keywords:** MIS-C, COVID-19, Vaccination, Children, Case report

## Abstract

**Background:**

Multisystem Inflammatory Syndrome in Children (MIS-C) is a rare complication, which develops within 3–6 weeks after SARS-CoV2 infection. The coronavirus disease 2019 (COVID-19) vaccine was firstly introduced in adults and adolescents and later in patients aged 5–11 years old. Although a reduced incidence of MIS-C and with less severe symptoms has been reported in vaccinated adolescents, there is little knowledge in children younger than 12 years of age. In addition, it is not understood whether MIS-C in vaccinated patients can be triggered by Covid19 vaccination or be secondary to a recent asymptomatic Sars-Cov2 infection.

**Case presentation:**

We describe the case of a Caucasian 6-year-old girl, one month after double COVID-19 vaccination, who presented fever, acute abdominal pain, rash, pharyngotonsillitis, cheilitis, cervical lymphadenopathy without a prior detected Sars-Cov2 infection. She also had lymphopenia, increase in inflammatory markers, cardiac and pulmonary involvement. Therefore, we dosed both anti Sars-Cov2 Spike and Nucleocapsid antibodies, which were positive and allowed us to confirm the diagnosis of MIS-C. We promptly administered intravenous immunoglobulins and methylprednisone, resulting in the initial regression of fever. During the hospitalization, the child also developed pancreatitis and severe neurological involvement, including irritability, drowsiness, distal tremor, dyskinesia and buccal asymmetry with complete resolution after 2 months. After 3 months from the onset of the symptoms, she reported a transient loss of hair compatible with telogen effluvium. After 12 months of follow-up, she did not show any symptomatic sequelae.

**Conclusions:**

This case raises the question of whether COVID-19 vaccination may be involved in the pathogenesis of MIS-C in children between the ages of 5 and 11 years old.

## Background

Since the end of December 2019, infection due to Sars-Cov2 virus has been responsible for a severe disease (Covid-19) with high morbidity and mortality worldwide which the World Health Organization declared pandemic in March 2020 [1, 2].

In children, Covid-19 occurred mainly in asymptomatic or paucisymptomatic forms [2–4]. However, from March 2020, the increase in “Kawasaki-like” cases temporally associated with SARS-Cov-2 infection has been observed all over the world. These symptoms were defined as Multisystem Inflammatory Syndrome in Children (MIS-C) or Pediatric Inflammatory Multisystem Syndrome (PIMS), rare complication which develops within 3–6 weeks of the primary infection [5, 6].

MIS-C was first reported in Italy and the UK but it is difficult to estimate its real incidence. The survey published by Dufort et al. in 2020 found an incidence of MIS-C in 2:100000 patients aged < 21 years old in the State of New York, whereas in the one by Payne et al., it was reported 5.1 per 100,000 person/month younger than 21 years [1, 7].

Neurological involvement in MIS-C is unexpectedly frequent (up to 35% of patients) and represented in most cases by headache, drowsiness, irritability, seizures and meningism that resolved without outcomes [8–11]. Rarely, significant neurological impairment is described [8–11]. Pancreatic involvement is very unusual as it is described in very few cases [12]. Moreover, there is currently little knowledge about Hair Loss After Sars-Cov2 Infection (HLASCI). A transient loss of hair appears to be a late complication of MIS-C and it can present with different patterns [13–15]. Telogenen Effluvium (TE) is the most frequently described and is characterized by a homogeneous reduction of hair volume in the absence of visible areas of alopecia [13–15].

Early treatment is essential and the first line of therapy is represented by intravenous immunoglobulin to achieve the anti-inflammatory effect [7, 16].

Covid-19 vaccine was firstly introduced in adults and adolescents and, at the end of 2021 in patients aged 5–11 years old [17]. Since then, a reduced incidence of MIS-C and with milder symptoms has been reported principally in adolescents [17]. Nowadays, there is little knowledge on a possible protective effect of Sars-Cov2 vaccination towards MIS-C in children aged 5–11 years old [17].

In addition, it is not clear if Covid19 vaccination plays a role in the onset of MIS-C in vaccinated children or if it is due to an asymptomatic Sars-Cov2 infection [17]. Few cases are described about this possible correlation and they involve principally adolescents. Among the episodic reports on children < 12 years old, we present the case of a 6-year-old girl, the youngest to our knowledge who received 2 doses of Covid19 vacine and developed severe MIS-C with extensive involvement.

## Case presentation

A Caucasian 6-year-old girl with no prior medical history, came to our attention due to 48 h fever, acute abdominal pain, lymphopenia (L 380/ul) and increase in inflammatory markers: erythrocyte sedimentation rate (ESR 37 mm/h with normal values 1–18), C-reactive protein (CRP: 225.2 mg/L with normal value < 2), procalcitonin (PCT: 24 ng/ml with normal value < 0.5). She was hospitalized in the Pediatric Surgery Unit in the suspicion of acute appendicitis where antibiotic therapy was started.

Given the persistence of her symptoms and the appearance of urticarial rash, pharyngotonsillitis, cheilitis, cervical lymphadenopathy and pain on mobilization of the head, she was transferred to our Pediatric Unit. We performed blood, urine and stool cultures that resulted negative. The rectal swabs excluded viral gastrointestinal infections; the nasal and pharyngeal swabs were negative for respiratory pathogens, included Sars-Cov2 and beta hemolytic streptococcus. Serology for TORCH complex and Epstein-Barr Virus showed negative results. We also dosed and found an increase in troponin (38.5 ng/L, normal value < 0.5 ng/L), proBNP2 (25600 pg/ml, normal value < 300), D-dimers 3081 ng/ml (normal value < 500), Interleukin-6 (692.50 pg/ml, normal value 0.5–6.40).

**Fig. 1:**
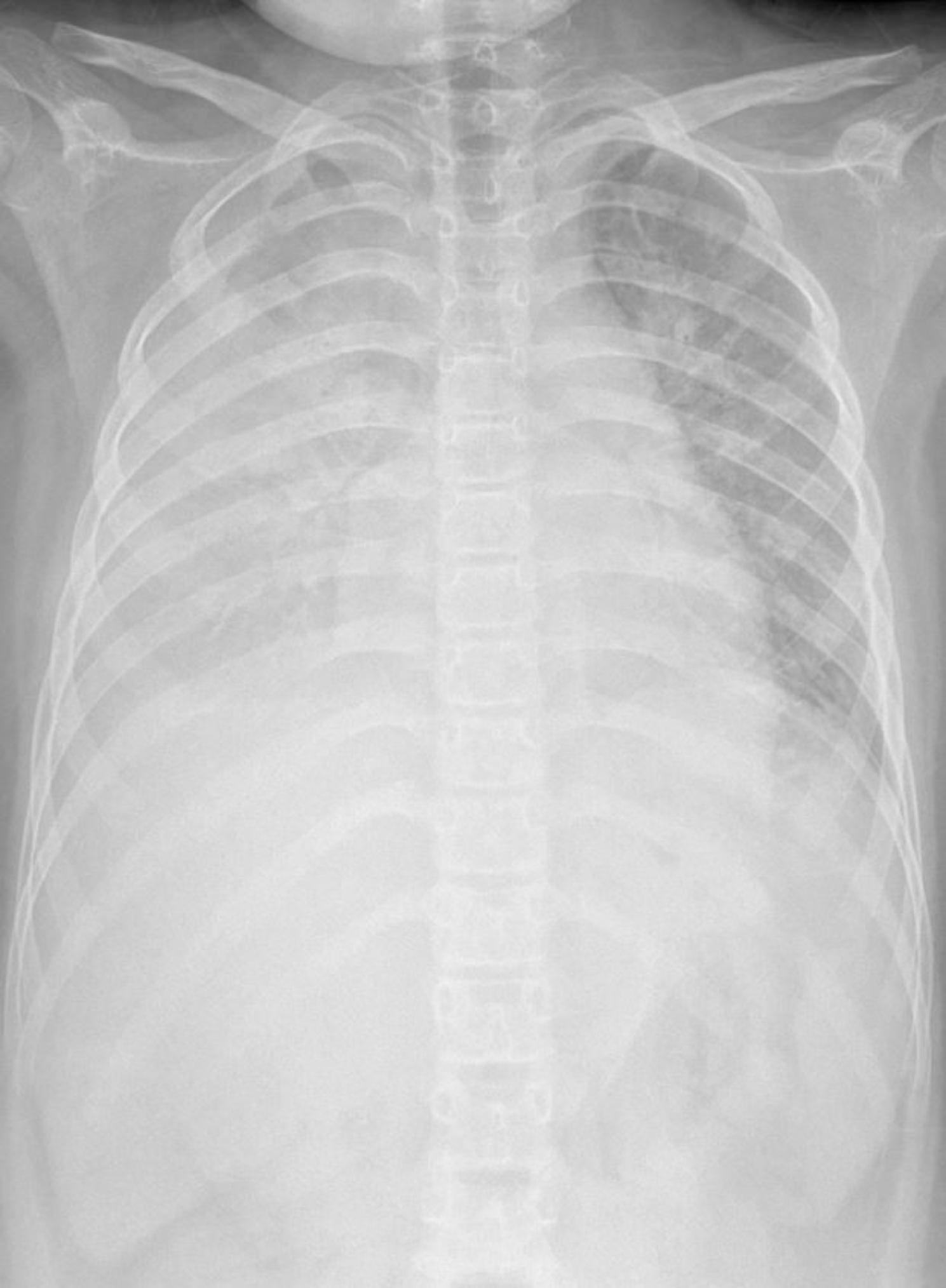
Chest radiography

**Fig. 2:**
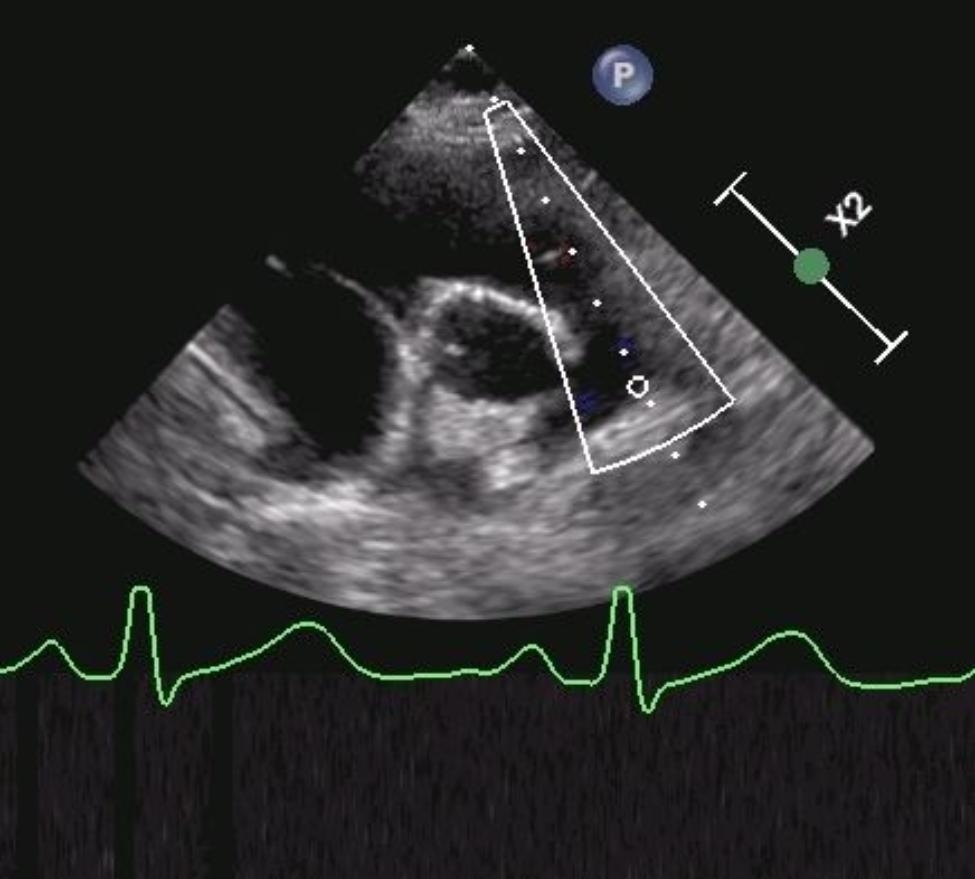
Echocardiography showing mild right sections dilatation

Chest x-ray showed pleural effusion, consolidation of the underlying pulmonary parenchyma, interstitial thickening of the left hemithorax [Fig. 1]. Echocardiography described a slightly reduced left ventricular systolic function and right sections dilatation, slight mitral regurgitation in the absence of aneurysmal dilatation of the coronary arteries or pericardial effusion [Fig. 2].

In the suspicion of MIS-C, we dosed anti Sars-Cov2 spike protein (anti-S) antibodies, which were positive. However, the patient received Covid-19 vaccination one month before the admission and did not report a detected Sars-Cov2 infection. Thus, we dosed anti Sars-Cov2 Nucleocapsid (anti-N) antibodies that were compatible with a status of previous asymptomatic Sars-Cov2 infection allowing us to confirm the diagnosis of MIS-C. We administered intravenous immunoglobulins (2 g/kg) and methylprednisone (30 mg/kg/day for 3 days, then 2 mg/kg/day), associated with proton pump inhibitors, broad-spectrum antibiotics (pipericillin/tazobactam), antiplatelet agents and enoxaparin due to the procoagulant risk associated with the patient.

Since day 4 of the hospitalization, fever disappeared and she showed improvement in general conditions. Inflammatory markers started decreasing, whereas amylase and lipase showed a gradual elevation for which the patient underwent an abdominal ultrasound detecting an enlarged pancreas without changes in the parenchyma.

On day 6, she became irritable, uncooperative and sleepy although awakenable and developed dyskinesia. The cerebellar tests found motor clumsiness. Distal tremor was detected through Mingazzini test and there was a slight asymmetry of the buccal fissure.

**Fig. 3:**
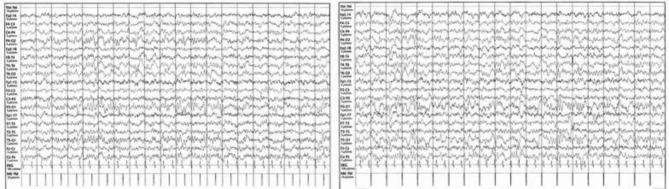
EEG with diffuse slow activity and sharp anomalies on the right temporo-parieto-occipital site

**Fig. 4:**
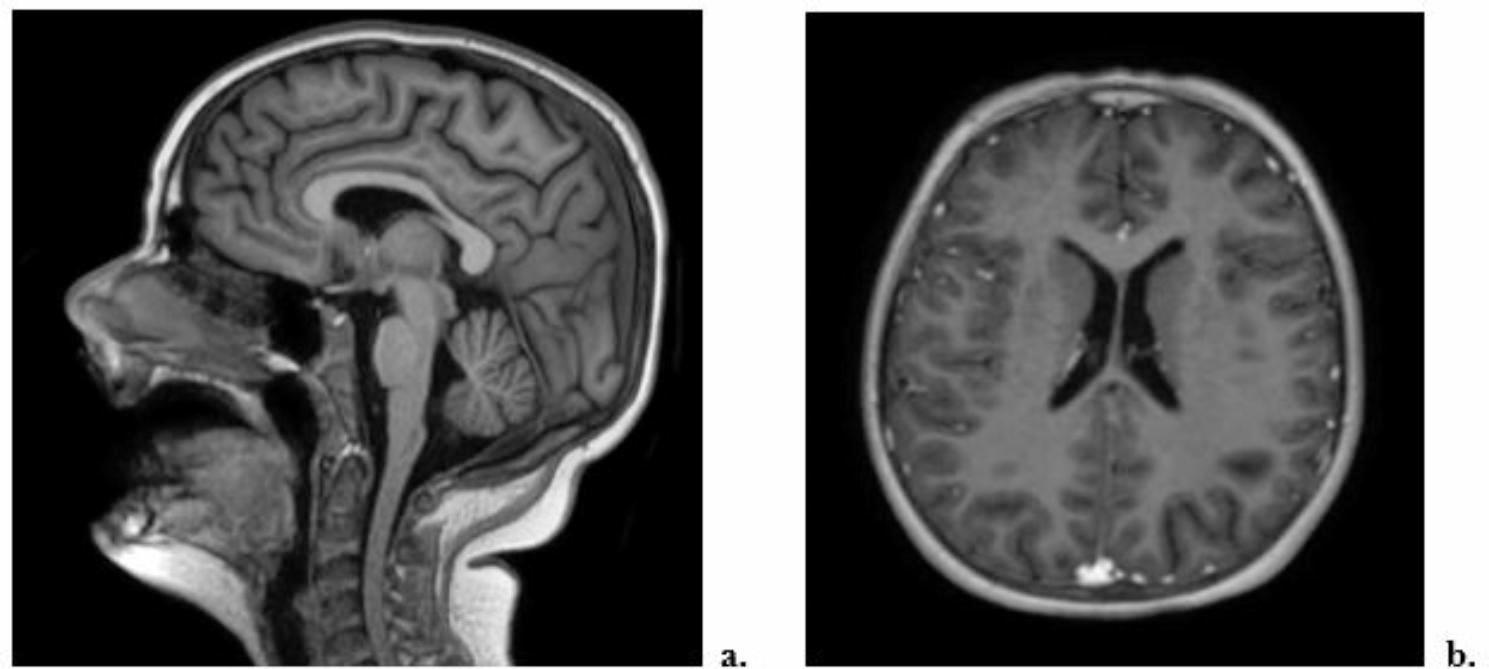
Brain MRI: **a**. sagittal view; **b**: axial view

The EEG detected a diffuse slow and pseudo-periodic activity mixed with sharp anomalies on the right temporo-parieto-occipital site, slowed background rhythm [Fig. 3]. Brain and spinal cord MRI with paramagnetic contrast resulted negative [Fig. 4]. On day 9, we started to observe the improvement of her neurological symptoms and EEG, whereas the pancreatic function reached a peak (amylase 293 U/L and lipase 500 U/L). During the following days, we obtained the normalization of all laboratory tests and of chest x-ray, echocardiography and abdomen echography. Therefore, on day 19 the patient was discharged with a strict follow-up. After two months, the patient presented the complete regression of her neurologic symptoms and normalization of EEG. However, three months after the onset, she reported unusual complete hair loss compatible with TE with complete resolution after three other months. After 12 months of follow-up, our patient did not show any symptomatic sequelae.

## Discussion and conclusions

MIS-C is a severe complication of Sars-Cov2 infection. According to the World Health Organization, it is characterized by persistent fever for more than 3 days associated with previous symptomatic or asymptomatic Sars-Cov2 infection demonstrable by serology or nasopharyngeal swab in the absence of other infectious diseases [9].

The diagnosis requires the elevation of laboratory signs of inflammation and multi-organ involvement [9, 12, 18]. The most frequent manifestations are fever, gastrointestinal symptoms and abdominal pain sometimes initially misdiagnosed as appendicitis or peritonitis [19]. Other common manifestations are cardiac, erythema and rash and non-purulent conjunctivitis; respiratory symptoms are less usual [19]. Children with MIS-C can present with central and/or peripheral nervous system symptoms with an unexpected high incidence (up to 35%), the most frequent being headache, acute encephalopathy and asthenia [10, 11, 19–22]. Hair loss appears to be another late complication of MIS-C with Telogenen Effluvium (TE) being the most frequently described [11, 13–15, 23]. Prompt diagnosis and treatment are fundamental. Intravenous immunoglobulins are the first line drugs [7, 16]. Other therapies include corticosteroids, aspirin, broad spectrum antibiotic coverage pending the results of the culture tests, gastric protectors and, in refractory cases, biologics (anakinra, tocilizumab, or infliximab) [24–29]. Mortality for MIS-C is around 1% in high-income countries and up to 9% in developing countries [17].

Our patient progressively developed multi-organ involvement starting with abdominal pain, cutaneous rash, cardiac and respiratory involvement. She also presented acute pancreatitis, a rare manifestation that was reported in 3% of patients in the case series published by Feldstein et al. and in other episodic cases [30]. Differently from the case by Stevens et al., our patient showed an increase in pancreatic enzymes after 4 days of hospitalization and not at the onset of the disease [31].

Neurological symptoms appeared after 6 days from the onset of fever and persisted for almost two months in contrast with the international literature where complete regression was observed after 10 days [9, 22].

In their retrospective analysis, Bova et al. found that 46.7% of patients with MIS-C showed signs of altered mental status such as irritability, agitation, apathy and mood and behavioral changes and 35.4% focal neurological signs including dyskinesia, muscle tone alterations and abnormal reflexes [20]. In line with these findings, in our patient the first neurological manifestations were irritability and drowsiness, followed by distal tremor, dyskinesia, motor clumsiness and buccal asymmetry.

Moreover, she did not show any cognitive, psychological and behavioral sequelae in her follow-up, differently from what observed in previous studies [11, 20, 22].

A lumbar puncture with analysis of the cerebrospinal fluid, EEG and brain MRI are normally required. Nevertheless, the majority of patients undergo only to an EEG whose principal features are focal or generalized slowing and epileptic abnormalities, both observed in our patient with normalization after 2 months, supporting the gravity of her clinical picture [9, 10, 20].

Hair loss appears to be another late complication of MIS-C [11, 13–15]. Telogenen Effluvium (TE) is the most frequently described nonscarring hair loss characterized by a homogeneous reduction of hair volume without alopecia. Potential triggers for TE are infections, systemic diseases, like in our case, severe emotional stress, major surgery, rapid weight loss, nutritional deficiency, endocrine disorders and drug exposure [23]. Acute TE occurs within 2–3 months after the triggering event and usually resolves spontaneously or when the trigger is removed [14, 32, 33].

A direct damage to the hair follicle and/or indirect damage mediated by the cytokine storm is suspected. Interleukin-6, a pro‐inflammatory cytokine involved during MIS-C, may play a role as it is suspected to predispose and exacerbate hair loss by inhibiting hair shaft elongation and hair follicle proliferation [34].

A post infectious immune response seems to be the principle determinant for MIS-C. The triggered “cytokine storm”, represented by an abnormal production of TNF alpha, IL-6 and IL-1, is responsible for a hyperinflammatory state that leads to endothelial damage and finally to multiple organ dysfunction [35, 36]. This hyperinflammatory state is also observed in Kawasaki syndrome and macrophage activation syndrome/hemophagocytic lymphohistiocytosis, thus their clinical manifestations overlap [10, 19, 35]. The presentation of MIS-C is also very similar to toxic shock syndrome (TSS). In this case, a superantigenic pathomechanism is considered responsible for uncontrolled cytokines release [35, 37]. In fact, Rivas et al. found a structure of Sars-Cov2 spike 1 glycoprotein with high resemblance to Staphyloccoccal enterotoxin B, cause of TSS [38].

MIS-C clinical features should also be differentiated from severe systemic bacterial infections, viral infections, severe allergic reactions such as Stevens-Johnson syndrome, toxic epidermal necrolysis or drug reaction with eosinophilia and systemic symptoms (DRESS), and multiple organ dysfunction syndrome (MODS) [37]. The latter can occur with numerous critical conditions and can be caused by different mechanisms, mostly a severe, systemic inflammatory process [39].

The incidence of MIS-C has decreased since its first appearance. A recent study collected data from four countries across two continents in order to analyze the different patients’ characteristics during Alpha, Delta and Omega Sars-Cov2 variants [40]. They found that patients with MIS-C were more frequent during Alpha wave, but they were younger and less sick during the Delta and Omega waves, consistent with other studies [2, 36, 41–43].

This could be the result of a decrease in virus pathogenicity, a preference for younger children for the last variants, a diverse immune response of children or the vaccination of adolescents ≥ 12 years old occurred during the Delta wave [2, 8, 17, 40, 44]. In fact, a study conducted in South Africa among unvaccinated children reported that MIS-C occurred in all age groups without any difference among virus waves [40].

Various studies demonstrated a reduction in MIS-C after the introduction of Sars-Cov2 vaccine. In fact, a US multicenter case-control investigation showed that MIS-C was 84% less probable in children ≥ 5 years old after two doses of Pfizer-BioNTech vaccine [45]. Another French population-based study revealed that MIS-C affected mostly unvaccinated patients, whereas in children with at least one dose of vaccine, its incidence was reduced by 91% [46]. Moreover, during the Delta wave, the Pfizer-BioNTech vaccine was found to be protective for MIS-C in 94% of children [17]. A recent systematic review and meta-analysis supported these findings, although mostly in children aged ≥ 12 years old, and highlighted the importance of mRNA COVID-19 vaccine in all eligible patients [17].

Vaccinated children can still develop MIS-C although less frequently and in milder forms, as demonstrated by the 20% higher rate of ICU admission of unvaccinated patients [35, 47].

Differently from what observed in adolescents, our 6-year old patient developed a severe presentation of MIS-C with extensive multiorgan involvement even though she received two doses of Sars-Cov2 vaccine. Moreover, as she did not report a prior detected Sars-Cov2 infection, anti-N IgG antibodies played a fundamental role. In fact, COVID-19 vaccination determines an immune response with the rise of anti-S antibodies that therefore are not useful to discriminate a prior infection [48]. On the contrary, anti-N antibodies rise only after Sars-Cov2 infection with a progressive decrease after 2 months and therefore are essential for immunized and asymptomatic patients [48, 49].

Nowadays, it is still not understood whether MIS-C can be secondary to vaccination or to a recent and often not recognized Sars-Cov2 infection [17]. Cases of vaccine-induced MIS (MIS-V), defined in the absence of prior Sars-Cov2 infection, are exceptional [35, 48, 50]. The US national vaccine passive surveillance system reported 79 cases of MIS-C following Covid19 vaccination in the period from 1 January 2020 to 31 December 2022. Among these patients, 79.7% were aged between 6 and 15 years old. However, it was not possible to establish whether MIS-C was caused by the vaccine or a recent Sars-Cov2 infection or other inflammatory conditions [50]. Cortese et al. conducted a surveillance study from November 2021 to March 2022 for MIS-C occurring within 90 days from Sars-Cov2 immunization in patients aged 5–11 years old [51]. They reported 58 children with history of Covid19 and 4 children with no proof of Sars-Cov2 infection. Other surveillance studies among United States adolescents and from France, found that the majority of vaccinated adolescents with MIS-C had evidence of a prior Sars-Cov2 infection. The other few cases may be due to different inflammatory diseases or false negative anti-N antibodies [17, 51].

In addition, the delay between Sars-Cov2 vaccination and MIS-C onset is reported shorter than Covid19 and MIS-C and it seems that MIS-V manifest with milder symptoms [17, 50–52].

Few cases of MIS-C in vaccinated children have been reported and mostly of adolescents [Table 1]. In particular, Zambrano et al. described only 5 cases of MIS-C in adolescents previously vaccinated with 2 doses [45]. Dejong et al. published a case of MIS-C in a vaccinated adolescent with sickle cell disease, whereas Consolini et al. the case of a fully vaccinated 17-year-old-girl who developed MIS-C with fever, cutaneous, respiratory, hepatic and pancreatic involvement [48, 60].

**Table 1:** Case reports of MIS-C in vaccinated children

Authors	Number of patients	Patient age (years)	Vaccine	Number of doses	Days from vaccine to onset	Sars-Cov2 PCR test positive	Atibodies anti-Spike	Atibodies anti-Nucleocapsid	Covid19
Haq K et al. [53]	1	5	Pfizer-BioNTech	1	15	yes	NA	positive	symptomatic
Demharter NS et al. [54]	1	9	Pfizer-BioNTech	2	31	yes	not tested	not tested	asymptomatic
Saeed S et al. [55]	1	11	Vero Cell	2	5	no	positive	not tested	no
Yalçınkaya R et al. [56]	1	12	Pfizer-BioNTech	1	27	no	positive	negative	no
Abdelgalil AA et al. [57]	1	12	Pfizer-BioNTech, then Moderna	2	20	no	positive	not tested	no
Poussaint TY et al. [58]	1	12	Pfizer-BioNTech	2	2	no	positive	negative	no
Lee S et al. [59]	1	13	Pfizer-BioNTech	2	91	yes	NA	NA	asymptomatic
DeJong J et al. [60]	1	14	Pfizer-BioNTech	2	60	no	NA	positive	asymptomatic
Hugh McGann P et al. [61]	1	16	Pfizer-BioNTech	1	12	no	positive	negative	no
Consolini R et al. [48]	1	17	Pfizer-BioNTech	2	120	no	positive	not tested	symptomatic
Chai Q et al. [62]	1	17	Pfizer-BioNTech	2	5	no	positive	negative	no
Karatzios C et al. [63]	2	12; 14	Pfizer-BioNTech; Pfizer-BioNTech	1; 1	35; 30	no; no	positive; positive	negative; negative	no; no
Collignon C et al. [64]	2	12; 15	Pfizer-BioNTech; Pfizer-BioNTech	1; 1	4; 4	no; no	positive; positive	negative; negative	symptomatic; NA
Jain E et al. [65]	2	15;17	Pfizer-BioNTech; Pfizer-BioNTech	1; 1	6; 7	no; no	negative; positive	positive; not tested	NA; NA

Legend: NA: Not Available

The majority of children developed symptoms within 30 days from Covid19 vaccination. Only 3 patients had history of previous symptomatic Sars-Cov2 infection and anti-N antibodies were found in other 3 children resulting in a prior asymptomatic infection.

Among the only 3 reported cases of MIS-C in vaccinated children aged < 12 years, Demharter et al. portrayed a case of a 9-year-old girl who received two doses of Pfizer-BioNTech Covid19 vaccine 30 days before developing MIS-C [54]. She had a Sars-Cov2 PCR test for travel purposes while asympomatic that resulted positive 16 days before the onset of her clinical manifestations characterized by fever, gastrointestinal manifestations and prolonged thrombocytopenia [54].

In line with these findings, our patient developed MIS-C symptoms 30 days after the second dose of Covid19 vaccine, therefore not completely excluding its possible culpability, and antibodies anti-N enabled the diagnosis of recent asymptomatic Covid19. Nonetheless her phenotype was severe with extensive multiple organ involvement.

Our case is among the episodic patients < 12 years old described in the literature and the youngest girl to our knowledge who developed MIS-C after 2 doses of vaccine with a chronology of events that cannot exclude vaccination’s involvement in its pathogenesis.

In conclusion, prompt recognition of MIS-C remains crucial in order to correctly treat the patients even though its incidence has decreased. Sars-Cov2 vaccination in patients aged > 12 years old played a protective role on the onset of MIS-C as well as on its severity. Currently, there are not large studies on the effects of COVID-19 vaccination in young children < 12 years old. Moreover, there is not clear evidence of a possible culpability of Covid19 vaccine for the onset of MIS-C. Only extremely rare cases are described in this age group. We believe that our case may be an input for further research about the role of Covid19 vaccine towards MIS-C in younger children.

## Data Availability

The data used and analysed during the current study are available from the corresponding author on reasonable request.

## Abbreviations

MIS-C: Multisystem Inflammatory Syndrome in Children
COVID-19: Coronavirus disease 2019
HLASCI: Hair Loss After Sars-Cov2 Infection
TE: Telogenen Effluvium
ESR: Erythrocyte sedimentation rate
CRP: C-reactive protein
PCT: Procalcitonin
Anti-S: Anti-Spike protein
Anti-N: Sars-Cov2 Nucleocapsid
ADEM: Acute Disseminated Encephalomyelitis
TSS: Toxic shock syndrome
DRESS: Drug Reaction with Eosinophilia and Systemic Symptoms
MODS: Multiple organ dysfunction syndrome
